# Dietary Niacin Intake and Mortality Among Individuals With Nonalcoholic Fatty Liver Disease

**DOI:** 10.1001/jamanetworkopen.2023.54277

**Published:** 2024-02-01

**Authors:** Jie Pan, Yujia Zhou, Nengzhi Pang, Lili Yang

**Affiliations:** 1 Guangdong Provincial Key Laboratory of Food, Nutrition and Health, Department of Nutrition, School of Public Health, Sun Yat-sen University, Guangzhou, China

## Abstract

**Importance:**

Evidence regarding the effect of dietary niacin intake on the risk of mortality among patients with nonalcoholic fatty liver disease (NAFLD) is scarce.

**Objective:**

To examine the association of dietary niacin intake with all-cause mortality and cardiovascular disease (CVD) mortality among individuals with NAFLD.

**Design, Setting, and Participants:**

This cohort study used data from the National Health and Nutrition Examination Survey (2003-2018). In total, 4315 adults aged 20 years or older with NAFLD were included, with NAFLD defined using the United States Fatty Liver Index.

**Exposure:**

Dietary niacin intake levels.

**Main Outcomes and Measures:**

Weighted Cox proportional hazards models and restricted cubic splines were used to estimate hazard ratios and 95% CIs for all-cause and CVD mortality. Data were analyzed March 1 to September 1, 2023.

**Results:**

This cohort study included data from 4315 participants in the analysis (mean [SD] age, 52.5 [16.2] years; 1670 participants ≥60 years [weighted, 30.9%]; 2351 men [weighted, 55.0%]). During a median (IQR) follow-up of 8.8 (4.6-11.8) years, 566 deaths were recorded, of which 197 were attributed to CVD. Compared with participants with a niacin intake of 18.4 mg or lower (the lowest tertile), the multivariable-adjusted hazard ratios for participants with a niacin intake of 26.7 mg or higher (the highest tertile) were 0.70 (95% CI, 0.50-0.96) for all-cause mortality (*P* = .03 for trend) and 0.65 (95% CI, 0.35-1.20) for CVD mortality (*P* = .16 for trend).

**Conclusions and Relevance:**

Findings from this cohort study suggest that higher dietary niacin intake may be associated with lower risk of all-cause mortality among individuals with NAFLD. There was no evident inverse association between dietary niacin intake and the risk of CVD mortality.

## Key Points

**Question:**

Is higher dietary niacin intake associated with lower risk of all-cause mortality and cardiovascular disease mortality among individuals with nonalcoholic fatty liver disease (NAFLD)?

**Findings:**

In this cohort study of 4315 patients with NAFLD, higher dietary niacin intake was associated with lower risk of all-cause mortality. However, there was no evident inverse association between dietary niacin intake and the risk of cardiovascular disease mortality.

**Meaning:**

The findings suggest that patients with NAFLD may benefit from increased dietary niacin intake.

## Teaser

This cohort study analyzes data from the National Health and Nutrition Examination Survey to assess whether there is an association between dietary niacin intake and all-cause mortality or cardiovascular disease mortality among individuals with nonalcoholic fatty liver disease.

## Introduction

Nonalcoholic fatty liver disease (NAFLD) is a global public health problem with an estimated prevalence of 32.4% worldwide and 47.8% in the US.^1^ In 2016, the overall mortality among patients with NAFLD was reported to be 15.4 per 1000 person-years,^2^ with the number of deaths among these patients doubling in the last 3 decades globally.^3^ Cardiovascular disease (CVD) is the primary cause of death among patients with NAFLD.^4^ Thus, it is particularly important to reduce the burden of disease and the risk of NAFLD mortality.

The role of nutrition in promoting health has received substantial attention. Niacin, also known as vitamin B_3_, acts as a precursor in the synthesis of the pyridine coenzymes nicotinamide adenine dinucleotide (NAD) and nicotinamide adenine dinucleotide phosphate (NADP), which play essential roles in a wide range of cell metabolic reactions, energy metabolism, and redox reactions.^5,6^

Although niacin supplementation to increase NAD levels has been found to have a modest remission effect on fatty liver in an animal study^7^ and to be associated with improved muscle performance in a human intervention study,^8^ no prospective studies have been conducted assessing the association between niacin intake levels from foods and the risk of mortality in patients with NAFLD. To address this research gap, our study examined the associations of dietary niacin intake with the risks of all-cause mortality and CVD mortality among individuals with NAFLD, using a nationally representative sample of adults in the US.

## Methods

### Study Design and Population

This cohort study used data from the National Health and Nutrition Examination Survey (NHANES), an examination program conducted to evaluate the health and nutritional status of populations in the US. The survey gathers information on a biennial basis using a complex, multistage probability sampling approach from the noninstitutionalized civilian population of the US.^9^ The protocols of the NHANES study were approved by the Institutional Review Board of the National Center of Health Statistics. All participants provided informed written consent at enrollment. This study followed the Strengthening the Reporting of Observational Studies in Epidemiology (STROBE) reporting guideline for cohort studies.

**Figure 1.**
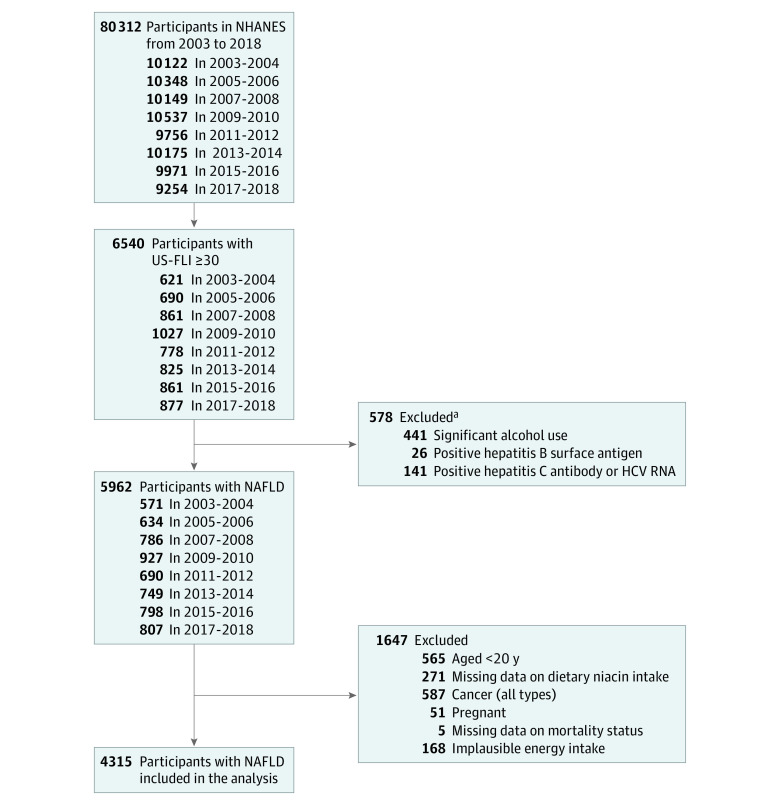
Flowchart Illustrating Selection of the Study Population in NHANES From 2003 to 2018 HCV indicates hepatitis C virus; NAFLD, nonalcoholic fatty liver disease; NHANES, National Health and Nutrition Examination Survey; and US-FLI, United States Fatty Liver Index. ^a^The number of exclusions exceeds the total (578) because some participants met more than 1 exclusion criterion.

We used data from US adults aged 20 years or older who participated in 8 cycles (2003-2004 to 2017-2018) of NHANES. Among the 80 312 participants in NHANES from 2003 to 2018, 6540 had a US Fatty Liver Index (FLI) of 30 or higher (Figure 1). After excluding participants with significant alcohol consumption or who tested positive for hepatitis B surface antigen, hepatitis C antibody, or hepatitis RNA, the remaining participants were considered to have NAFLD. Participants who were younger than 20 years, pregnant, had any type of cancer, had missing data on dietary niacin intake or mortality status, or had an implausible energy intake (<600 or >3500 kcal/d for women and <800 or >4200 kcal/d for men)^10^ were further excluded.

### Measurement of Dietary Niacin Intake

The dietary interview component, known as What We Eat in America, was conducted with a standardized Automated Multiple-Pass Method (AMPM) in collaboration between the US Department of Agriculture (USDA) and the US Department of Health and Human Services. The AMPM was designed to provide an efficient and accurate means of collecting intakes for large-scale national surveys, and several studies have reported on the reliability of the AMPM method.^11,12,13^ All eligible NHANES participants were given two 24-hour dietary recall interviews to report the types and quantities of foods they consumed in the 24 hours prior to the interview (from midnight to midnight). One dietary recall was conducted in person at the Mobile Examination Center, while the second recall was conducted by telephone interview approximately 3 to 10 days after the first recall. Nutrients and food components of all foods were calculated using the USDA Food and Nutrient Database for Dietary Studies.^14^ The mean of the participants’ 2 dietary recalls, or the value of 1 of them (if participants had only 1 recall), was used as the daily dietary niacin intake in this study. Of the 4315 participants, 335 (7.8%) participants had only 1 dietary recall.

### Definition of NAFLD

Fatty liver was defined using the FLI calculated as follows:

FLI = e^y^ / (1+e^y^) × 100, where

y = (−0.8073 × non-Hispanic Black + 0.3458 × Mexican American) + (0.0093 × age) + (0.6151 × log_e_ γ glutamyltransferase) + (0.0249 × waist circumference) + (1.1792 × log_e_ insulin) + (0.8242 × log_e_ glucose) − 14.7812,

with values for non-Hispanic Black and Mexican American assigned 1 if the participant belonged to that ethnicity, otherwise 0; age measured in years; γ glutamyltransferase in international units per liter; waist circumference in centimeters; insulin in picomoles per liter; and glucose in milligrams per deciliter.^15^ Using the recommended value, an FLI of 30 or higher was selected to define fatty liver.^16,17,18,19^ This definition had shown an area under the receiver operating characteristic curve of 0.80 (95% CI: 0.77-0.83) in predicting ultrasonographically determined NAFLD.^15^ We further defined NAFLD as having an FLI of 30 or higher, excluding other known causes of chronic liver disease. Those causes included viral hepatitis (indicated by a positive test result for hepatitis B surface antigen, hepatitis C antibody, or hepatitis C RNA) and significant alcohol consumption (≥2 drinks per day for men or ≥1 drink per day for women; a drink was defined as 14 g of pure alcohol^20,21^).

### Ascertainment of Mortality

Data on follow-up and mortality status were obtained by linking the NHANES data to the National Death Index records through December 31, 2019. All-cause mortality refers to death from any cause. We determined the underlying cause of death by referring to codes from the *International Statistical Classification of Diseases and Related Health Problems, Tenth Revision* (*ICD-10*); CVD mortality was defined as death due to heart disease (codes I00-I09, I11, I13, and I20-I51) and cerebrovascular disease (codes I60-I69).

### Assessment of Covariates

Age was classified into 3 groups: 39 years or younger, 40 to 59 years, and 60 years or older. Race and ethnicity were categorized per patient-reported selections in NHANES as Mexican American, non-Hispanic Black, non-Hispanic White, and other (included other Hispanic, other non-Hispanic, and non-Hispanic multiple races) and were collected because they, together, were considered a confounder. Educational levels were categorized as less than high school, high school, and some college or above. The family income to poverty ratio was grouped into 3 categories: lower than 1.0, 1.0 to 3.0, and higher than 3.0. Smoking status was grouped into never smoker (defined as <100 cigarettes in a lifetime), current smoker (defined as ≥100 cigarettes in a lifetime), and former smoker (defined as ≥100 cigarettes and had quit smoking). Participants were classified as physically active if they had at least 150 minutes of moderate to vigorous physical activity per week; otherwise, they were classified as physically inactive according to the Centers for Disease Control and Prevention Physical Activity Guidelines for Americans. Body mass index (BMI, calculated as weight in kilograms divided by height in meters squared) was categorized as lower than 25.0, 25.0 to 29.9, and 30.0 or higher. Information on the use of dietary supplements during the past 30 days was collected by trained professionals. Dyslipidemia was defined as having at least 1 of the following conditions: total cholesterol concentration 200 mg/dL or higher (to convert to millimoles per liter, multiply by 0.0259), low-density lipoprotein cholesterol concentration 130 mg/dL or higher (to convert to millimoles per liter, multiply by 0.0259), triglyceride concentration 150 mg/dL or higher (to convert to millimoles per liter, multiply by 0.0113), or high-density lipoprotein cholesterol concentration below 40 mg/dL (to convert to millimoles per liter, multiply by 0.0259).^22^ Healthy Eating Index 2015 (HEI-2015), a diet pattern measure, was commonly used to evaluate diet quality according to the healthy eating pattern proposed by *Dietary Guidelines for Americans, 2015-2020*,^23^ and it was also recommended in *Dietary Guidelines for Americans, 2020-2025*.^24^ The scoring method for HEI-2015 is described in eTable 1 in Supplement 1; HEI-2015 scores ranged from 0 to 100, with higher scores corresponding to a healthier diet. Data on physician-diagnosed history of hypertension, high cholesterol, and diabetes were self-reported.

### Statistical Analysis

Given the complex sampling design of NHANES, all analyses in this study incorporate sample weights, strata, and primary sampling units. The weight was calculated based on the dietary day 1 sample weight divided by the number of cycles in the main analysis. When excluding participants with only 1 dietary recall, the weight was calculated based on the dietary day 2 sample weight divided by the number of cycles. Data are presented as unweighted frequencies (weighted percentages) for categorical variables and as medians (IQRs) for continuous variables. Differences among groups were compared using the Kruskal-Wallis test for continuous variables with nonnormal distribution and the χ^2^ test with the Rao and Scott second-order correction for categorical variables. Weighted Cox proportional hazards regression models were used to calculate hazard ratios (HRs) and 95% CIs to examine the associations between dietary niacin intake and the risk of all-cause mortality and CVD mortality. The proportional hazards assumption was tested using Schoenfeld residuals, and no violation was observed. Person-time was calculated from the NHANES interview date to the date of death or the end of the follow-up (December 31, 2019), whichever occurred first. Three models were constructed. Model 1 did not adjust for any covariate. Model 2 adjusted for age and sex. Model 3 additionally adjusted for race and ethnicity, educational levels, family income to poverty ratio, smoking status, BMI, total energy intake, diabetes, hypertension, dyslipidemia, use of dietary supplements, high-density lipoprotein cholesterol to total cholesterol ratio, and HEI-2015. To test for linear trends, a continuous variable was created by assigning a median value to each category.

Restricted cubic spline analysis with 4 knots (at the 5th, 35th, 65th, and 95th percentiles) was used to investigate the nonlinear associations between dietary niacin intake and both all-cause mortality and CVD mortality. The likelihood ratio test was conducted to assess nonlinearity. We further stratified the analyses by age, sex, race and ethnicity, educational levels, family income to poverty ratio, smoking status, BMI, diabetes, fibrosis-4 index and levels of vitamins B_1_, B_2_, B_6_, B_9_, and B_12_. The significance of the interactions was estimated using *P* values for the production terms between dietary niacin intake and the stratified factors.

We also conducted a series of sensitivity analyses: (1) to minimize the potential reverse causation bias, we excluded participants who died within 2 years of follow-up; (2) participants with only 1 dietary recall were excluded from the sensitivity analyses; (3) repeated analysis was conducted by excluding participants with NAFLD who died within 2 years of follow-up and had only 1 dietary recall; and (4) the number of knots of the restricted cubic spline was changed for repeated analysis using 3 knots (at the 10th, 50th, and 90th percentiles) and 5 knots (at the 5th, 27.5th, 50th, 72.5th, and 95th percentiles).

The laboratory methods used to measure glucose, insulin, alanine aminotransferase, aspartate aminotransferase, and γ glutamyltransferase levels changed during the NHANES data collection period, and we applied regression equations recommended by the National Center for Health Statistics to align those values over time. Covariates with missing values were imputed using the multiple imputation by chained equations method. All statistical analyses were performed from March 1 to September 1, 2023, using R software, version 4.2.3 (R Project for Statistical Computing). A 2-sided *P* < .05 was considered the threshold for statistical significance.

## Results

A total of 4315 confirmed cases of NAFLD were included in this study (mean [SD] age, 52.5 [16.2] years; 1670 participants ≥60 years [weighted, 30.9%]; 2351 men [weighted, 55.0%] and 1964 women [weighted 45.0%]); 1176 (13.9%) were Mexican American, 498 (6.3%) were non-Hispanic Black, 1822 (67.2%) were non-Hispanic White, and 819 (12.7%) were categorized as other. The baseline characteristics of 4315 participants with NAFLD according to tertile of dietary niacin intake (1440 participants in tertile 1 [≤18.4 mg]; 1441 participants in tertile 2 [18.5-26.6 mg]; and 1434 tertile 3 [≥26.7 mg]) are summarized in Table 1. Compared with participants in the lowest tertile of niacin intake, participants with higher dietary niacin intake were younger, more often male, had higher levels of education and family income, lower high-density lipoprotein cholesterol levels, and higher total energy intake, alanine aminotransferase, and aspartate aminotransferase levels. Similarities were found in the percentages of race and ethnicity, smoking status, physical activity, BMI, hypertension, high cholesterol, total cholesterol, and low-density lipoprotein cholesterol levels among the 3 groups.

**Table 1.** Baseline Characteristics of Participants With NAFLD in NHANES 2003 to 2018^a^

Characteristic	Patients, No. (%)				*P* value
		Tertile 1, ≤18.4, mg/d^b^	Tertile 2, 18.5-26.6, mg/d^b^	Tertile 3, ≥26.7, mg/d^b^	
Patients, No.	4315	1440	1441	1434	NA
Age, y					
≤39	1047 (27.2)	282 (24.5)	317 (24.0)	448 (32.2)	<.001
40-59	1598 (41.9)	436 (35.5)	558 (42.7)	604 (46.2)	
≥60	1670 (30.9)	722 (40.0)	566 (33.3)	382 (21.6)	
Sex					
Male	2351 (55.0)	524 (33.0)	766 (52.1)	1061 (75.3)	<.001
Female	1964 (45.0)	916 (67.0)	675 (47.9)	373 (24.7)	
Race and ethnicity					
Mexican American	1176 (13.9)	419 (14.5)	357 (12.3)	400 (14.8)	.10
Non-Hispanic Black	498 (6.3)	156 (5.9)	196 (7.4)	146 (5.5)	
Non-Hispanic White	1822 (67.2)	565 (65.1)	620 (67.8)	637 (68.3)	
Other^c^	819 (12.7)	300 (14.5)	268 (12.5)	251 (11.4)	
Educational level					
<High school	1397 (21.3)	583 (26.8)	434 (20.8)	380 (17.3)	<.001
High school	959 (24.2)	315 (25.9)	330 (23.8)	314 (23.3)	
Some college or above	1956 (54.5)	540 (47.3)	676 (55.4)	740 (59.4)	
Family income to poverty ratio					
<1.0	836 (14.5)	357 (21.7)	258 (12.8)	221 (10.4)	<.001
1.0-3.0	1785 (39.7)	612 (43.6)	578 (39.4)	595 (36.8)	
>3.0	1298 (45.8)	321 (34.6)	469 (47.9)	508 (52.8)	
Smoking status					
Never	2307 (52.4)	794 (52.2)	786 (53.1)	727 (51.8)	.44
Former	1277 (30.6)	413 (29.7)	418 (29.0)	446 (32.8)	
Current	729 (17.0)	232 (18.1)	236 (17.9)	261 (15.4)	
Physical activity					
Active	979 (54.8)	293 (55.9)	314 (52.5)	372 (55.9)	.63
Inactive	788 (45.2)	222 (44.1)	274 (47.5)	292 (44.1)	
BMI					
<25.0	177 (3.2)	76 (4.5)	59 (3.2)	42 (2.2)	.20
25.0-29.9	1067 (21.8)	366 (21.5)	348 (21.2)	353 (22.5)	
≥30.0	3057 (75.0)	988 (74.0)	1033 (75.6)	1036 (75.3)	
Total energy intake, median (IQR), kcal	2018 (1537-2573)	1447 (1177-1781)	1973 (1623-2379)	2596 (2152-3072)	<.001
Use of dietary supplements					
Yes	2072 (51.4)	717 (53.8)	688 (49.5)	667 (51.3)	.33
No	2242 (48.6)	722 (46.2)	753 (50.5)	767 (48.7)	
Hypertension					
Yes	2088 (47.7)	743 (49.2)	684 (47.0)	661 (47.1)	.71
No	2220 (52.3)	692 (50.8)	757 (53.0)	771 (52.9)	
High cholesterol					
Yes	1860 (49.0)	622 (48.8)	641 (48.8)	597 (49.4)	.97
No	1934 (51.0)	634 (51.2)	640 (51.2)	660 (50.6)	
Dyslipidemia					
Yes	3123 (73.6)	1029 (72.4)	1021 (70.5)	1073 (77.4)	.01
No	1177 (26.4)	408 (27.6)	413 (29.5)	356 (22.6)	
Diabetes					
Yes	1006 (19.6)	375 (22.2)	331 (19.8)	300 (17.4)	.03
No	3305 (80.4)	1063 (77.8)	1108 (80.2)	1134 (82.6)	
ALT, median (IQR), U/L	27 (20-36)	23 (18-33)	26 (21-36)	29 (22-39)	<.001
AST, median (IQR), U/L	25 (21-30)	24 (20-29)	24 (21-29)	25 (21-30)	<.001
TC, median (IQR), mg/dL	193 (166-221)	192 (165-222)	193 (167-221)	194 (166-220)	.88
TG, median (IQR), mg/dL	146 (104-209)	146 (102-198)	142 (103-208)	150 (106-214)	.11
LDL-C, median (IQR), mg/dL	114 (92-138)	113 (90-140)	116 (91-137)	114 (93-138)	.76
HDL-C, median (IQR), mg/dL	44 (38-52)	46 (39-54)	45 (38-52)	43 (37-51)	<.001
HEI-2015, median (IQR)	48 (39-58)	48 (38-59)	48 (41-58)	49 (40-57)	.60

Abbreviations: ALT, alanine aminotransferase; AST, aspartate aminotransferase; BMI, body mass index (calculated as weight in kilograms divided by height in meters squared); HDL-C, high-density lipoprotein cholesterol; HEI-2015, Healthy Eating Index 2015; LDL-C, low-density lipoprotein cholesterol; NA, not applicable; NAFLD, nonalcoholic fatty liver disease; NHANES, National Health and Nutrition Examination Survey; TC, total cholesterol; TG, triglyceride.

SI conversion factors: To convert ALT to microkatals per liter, multiply by 0.0167; TC, LDL-C, and HDL-C to millimoles per liter, multiply by 0.0259; TG to millimoles per liter, multiply by 0.0113.

^a^ All estimates accounted for complex survey designs, and all percentages are weighted.

^b^ Daily dietary niacin intake.

^c^ Other race and ethnicity includes other Hispanic, other non-Hispanic, and multirace individuals.

### Dietary Niacin Intake and All-Cause and CVD Mortality

**Figure 2.**
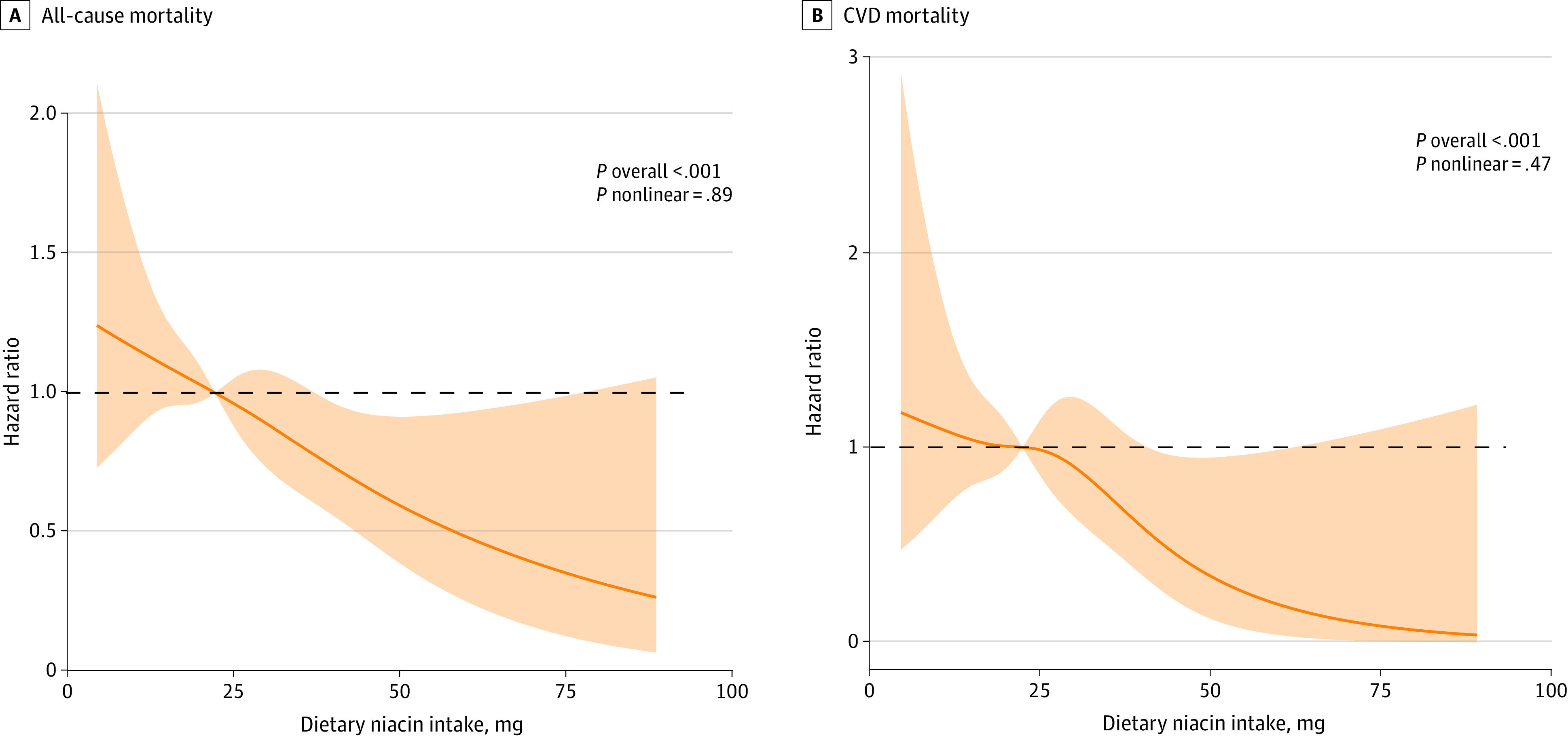
Association of Dietary Niacin Intake With All-Cause and CVD Mortality Among Individuals With NAFLD Hazard ratios were adjusted for age, sex, race and ethnicity, educational levels, family income to poverty ratio, smoking status, body mass index, total energy intake, diabetes, hypertension, dyslipidemia, dietary supplement, high-density lipoprotein cholesterol to total cholesterol ratio, and Healthy Eating Index 2015. Shaded areas represent 95% CIs. CVD indicates cardiovascular disease and NAFLD, nonalcoholic fatty liver disease.

During a median (IQR) follow-up of 8.8 (4.6-11.8) years, 566 deaths were recorded, of which 197 were attributed to CVD. In model 1, the HR for all-cause mortality in the third tertile was 0.47 (95% CI, 0.36-0.63) compared with the reference group (*P* < .001 for trend). After multivariable adjustment in model 3, the HR for all-cause mortality in the third tertile was 0.70 (95% CI, 0.50-0.96) compared with the reference group (*P* = .03 for trend). The E-value of 2.16 suggests that an unmeasured confounder must be associated with both the exposure (dietary niacin intake) and the outcome (mortality) with a relative risk greater than 2.16 to fully explain the current association. The HR for CVD mortality in model 1 in the third tertile was 0.42 (95% CI, 0.22-0.83) compared with the reference group (*P* = .008 for trend). The multivariable-adjusted HR for the middle category of dietary niacin intake (reference: lowest category of niacin intake, 1.00) was 0.87 (95% CI, 0.63-1.21), and for the highest it was 0.65 (95% CI, 0.35-1.20), for CVD mortality (*P* = .16 for trend) (Table 2). The dose-response associations between dietary niacin intake and all-cause and CVD mortality are shown in Figure 2. There was no evident nonlinear association between dietary niacin intake and either all-cause mortality (*P* = .89 for nonlinear) or CVD mortality (*P* = .47 for nonlinear) in the restricted cubic splines (both *P* < .001 for overall).

**Table 2.** Hazard Ratios for All-Cause and CVD Mortality Among Participants With NAFLD in NHANES 2003 to 2018

Model	Hazard ratio (95% CI)			*P* value for trend
	Tertile 1, ≤18.4 mg/d^a^	Tertile 2, 18.5-26.6 mg/d^a^	Tertile 3, ≥26.7 mg/d^a^	
**All-cause mortality**				
Total deaths, No.	242 of 1440	176 of 1441	128 of 1434	NA
Model 1^b^	1.00 [Reference]	0.73 (0.56-0.95)	0.47 (0.36-0.63)	<.001
Model 2^c^	1.00 [Reference]	0.72 (0.55-0.94)	0.55 (0.39-0.76)	<.001
Model 3^d^	1.00 [Reference]	0.78 (0.60-1.02)	0.70 (0.50-0.96)	.03
**CVD mortality**				
Total deaths, No.	94 of 1440	63 of 1441	36 of 1434	NA
Model 1^b^	1.00 [Reference]	0.80 (0.58-1.10)	0.42 (0.22-0.83)	.008
Model 2^c^	1.00 [Reference]	0.81 (0.58-1.13)	0.52 (0.28-0.97)	.03
Model 3^d^	1.00 [Reference]	0.87 (0.63-1.21)	0.65 (0.35-1.20)	.16

Abbreviations: CVD, cardiovascular disease; NA, not applicable; NAFLD, nonalcoholic fatty liver disease; NHANES, National Health and Nutrition Examination Survey.

^a^ Daily dietary niacin intake.

^b^ Crude model.

^c^ Adjusted for age and sex.

^d^ Further adjusted for race and ethnicity, educational levels, family income to poverty ratio, smoking status, body mass index, total energy intake, diabetes, hypertension, dyslipidemia, use of dietary supplements, high-density lipoprotein cholesterol to total cholesterol ratio, and Healthy Eating Index 2015.

### Stratified and Sensitivity Analyses

We found a significant interaction between dietary niacin intake and diabetes with the risk of all-cause mortality (*P* = .04 for interaction). For the subgroup with diabetes compared with the reference group (the first tertile), the HR of all-cause mortality in the third tertile was 0.82 (95% CI, 0.50-1.35). When the subgroup without diabetes was compared with the reference group, the HR of all-cause mortality in the third tertile was 0.58 (95% CI, 0.35-0.95). However, no significant interactions were found between dietary niacin intake and any other strata variables with the risk of all-cause mortality (Table 3). There were no significant interactions between dietary niacin intake and any strata variables with the risk of CVD mortality (eTable 2 in Supplement 1). There was a significant interaction between dietary niacin intake and vitamin B_6_ levels with the risk of all-cause mortality (*P* = .02 for interaction). For the subgroup with vitamin B_6_ levels lower than 1.7 mg, compared with the reference group (the first tertile), the HR of all-cause mortality in the third tertile was 0.26 (95% CI, 0.10-0.71). When the subgroup with vitamin B_6_ levels 1.7 mg or higher was compared with the reference group, the HR of all-cause mortality in the third tertile was 0.95 (95% CI, 0.59-1.55). No significant interactions were found between dietary niacin intake and any other B vitamins with the risk of all-cause mortality (eTable 3 in Supplement 1). In addition, there was no evident interaction between niacin and the fibrosis-4 index regardless of whether 1.30 or 2.67 was used as the cutoff value^25^ (eTable 4 in Supplement 1).

**Table 3.** Associations of Dietary Niacin Intake With All-Cause Mortality in Various Subgroups Among Participants With NAFLD in NHANES 2003-2018^a^

Characteristic	Hazard ratio (95% CI)			*P* value for interaction
	Tertile 1, ≤18.4 mg/d	Tertile 2, 18.5-26.6 mg/d	Tertile 3, ≥26.7 mg/d	
Age, y				
≤39	1.00 [Reference]	0.12 (0.03-0.47)	0.37 (0.12-1.10)	.57
40-59	1.00 [Reference]	0.90 (0.44-1.83)	0.69 (0.31-1.52)	
≥60	1.00 [Reference]	0.79 (0.57-1.09)	0.69 (0.47-1.01)	
Sex				
Male	1.00 [Reference]	0.81 (0.58-1.14)	0.63 (0.41-0.98)	.70
Female	1.00 [Reference]	0.82 (0.53-1.27)	1.15 (0.69-1.91)	
Race and ethnicity				
Mexican American	1.00 [Reference]	0.83 (0.42-1.67)	1.14 (0.52-2.49)	.44
Non-Hispanic Black	1.00 [Reference]	0.89 (0.42-1.92)	1.11 (0.50-2.45)	
Non-Hispanic White	1.00 [Reference]	0.72 (0.53-0.99)	0.60 (0.41-0.88)	
Other^b^	1.00 [Reference]	0.99 (0.45-2.20)	1.53 (0.56-4.14)	
Educational level				
<High school	1.00 [Reference]	0.81 (0.53-1.23)	0.92 (0.55-1.54)	.61
High school	1.00 [Reference]	1.01 (0.56-1.84)	1.01 (0.53-1.90)	
Some college or above	1.00 [Reference]	0.54 (0.33-0.89)	0.43 (0.24-0.79)	
Family income to poverty ratio				
<1.0	1.00 [Reference]	0.52 (0.30-0.91)	0.83 (0.44-1.58)	.30
1.0-3.0	1.00 [Reference]	0.70 (0.49-1.00)	0.58 (0.38-0.89)	
>3.0	1.00 [Reference]	1.24 (0.66-2.31)	1.05 (0.51-2.16)	
Smoking status				
Never	1.00 [Reference]	1.16 (0.69-1.94)	1.29 (0.72-2.31)	.32
Former	1.00 [Reference]	0.55 (0.36-0.84)	0.51 (0.30-0.87)	
Current	1.00 [Reference]	0.94 (0.44-2.01)	0.52 (0.22-1.24)	
BMI				
<25.0	1.00 [Reference]	1.69 (0.69-4.12)	2.49 (0.53-11.70)	.72
25.0-29.9	1.00 [Reference]	0.83 (0.44-1.54)	0.69 (0.34-1.40)	
≥30.0	1.00 [Reference]	0.79 (0.57-1.10)	0.77 (0.53-1.11)	
Diabetes				
Yes	1.00 [Reference]	0.60 (0.35-1.02)	0.82 (0.50-1.35)	.04
No	1.00 [Reference]	0.86 (0.61-1.19)	0.58 (0.35-0.95)	

Abbreviations: BMI, body mass index (calculated as weight in kilograms divided by height in meters squared); NAFLD, nonalcoholic fatty liver disease; NHANES, National Health and Nutrition Examination Survey.

^a^ Adjusted for age, sex, race and ethnicity, educational level, family income to poverty ratio, smoking status, body mass index, total energy intake, diabetes, hypertension, dyslipidemia, use of dietary supplements, high-density lipoprotein cholesterol to total cholesterol ratio, and Healthy Eating Index 2015; the strata variable was not included when stratifying by itself.

In sensitivity analyses, when excluding the participant who died within 2 years of follow-up, an inverse association between dietary niacin intake and all-cause mortality was found in models 1 and 2 (eTable 5 in Supplement 1). The associations of dietary niacin intake with all-cause and CVD mortality were not materially altered when participants with only 1 dietary recall were excluded (eTable 6 in Supplement 1). In addition, the results remained robust when repeated analysis was conducted by excluding participants with NAFLD who died within 2 years of follow-up and had only 1 dietary recall (eTable 7 in Supplement 1). The results of restricted cubic splines with 3 knots (at the 10th, 50th, and 90th percentiles) and 5 knots (at the 5th, 27.5th, 50th, 72.5th, and 95th percentiles) were consistent with the restricted cubic spline with 4 knots (eFigure 1 and eFigure 2 in Supplement 1).

## Discussion

To our knowledge, this large-scale, prospective cohort study is the first study to examine the association between dietary niacin intake and the risk of all-cause mortality and CVD mortality among individuals with NAFLD. We found that higher dietary niacin intake was associated with lower risk of all-cause mortality. After adjusting for multiple confounders, higher dietary niacin was not found to be associated with lower risk of CVD mortality. Stratified and sensitivity analyses demonstrated the robustness of the results we found.

Niacin is one of the precursors of NAD synthesis, which has become an important target for the prevention and treatment of liver disease. It has also been shown in population intervention studies that niacin can improve fatty liver and reduce liver fat content.^8,26^ In preclinical research, niacin has been found to inhibit and reverse hepatic steatosis and inflammation and to prevent fibrosis. These effects are achieved through the reduction of oxidative stress and inhibition of diacylglycerol acyltransferase 2 and NADPH oxidase activity as well as other possible mechanisms.^27,28^ In the present study, we also observed benefits associated with increased dietary niacin intake among patients with NAFLD. Specifically, a higher dietary niacin intake was associated with 30% (≥26.7 mg/d vs ≤18.4 mg/d) reduction in the risk of all-cause mortality among individuals with NAFLD. After adjustment for age and sex, higher niacin intake was associated with an approximately 50% reduction in the risk of CVD mortality among individuals with NAFLD, although this association was no longer evident after further adjustment for other potentially confounding variables. Existing evidence indicates that CVD has been identified as the primary cause of death among patients with NAFLD.^4^ Thus, it is crucial to extend the follow-up period in future studies, which will allow for a greater number of outcome events to be captured and will help to further investigate the association between dietary niacin intake and the risk of CVD mortality among individuals with NAFLD.

Niacin has been reported to increase glucose levels in individuals with diabetes.^29,30^ In addition, according to a randomized controlled trial study, 16-week treatment with extended release niacin (titrated from 0.5 g per week to a final dose of 2 g per week during the first 3 weeks) in patients with NAFLD significantly increased fasting glucose and insulin levels and homeostasis model assessment of insulin resistance.^31^ Apart from those intervention studies, an observational study also found that patients with NAFLD combined with diabetes had significantly higher risk (60%) of all-cause mortality and 41% higher risk of CVD mortality than patients with NAFLD without diabetes.^32^ In the present study, when the subgroup with diabetes was compared with the reference group, the HR for all-cause mortality was 0.82 (95% CI, 0.50-1.35) in the third tertile, and when the subgroup without diabetes was compared with the reference group, the HR for all-cause mortality was 0.58 (95% CI, 0.35-0.95) in the third tertile (*P* = .04 for interaction). Considering the effects of niacin in patients with diabetes and the effects of diabetes itself in patients with NAFLD, combined with the results of our subgroup analysis, it is suggested that higher niacin intake may be more strongly associated with lower risk of mortality in patients with NAFLD but without diabetes.

Another notable finding of this study is that in the subgroup with vitamin B_6_ levels lower than 1.7 mg, the HR of all-cause mortality was 0.26 (95% CI, 0.10-0.71) in the third tertile when compared with the reference group (the first tertile). This finding suggests that patients with NAFLD with vitamin B_6_ intake lower than 1.7 mg/d may benefit more from niacin. This finding aligns with existing theories and is biologically plausible. Vitamin B_6_ is required for the biosynthesis and metabolism of niacin, and low levels of vitamin B_6_ reduce the conversion of tryptophan to niacin,^33^ making it even more important to increase dietary niacin intake.

The potential mechanism underlying the association between dietary niacin intake and mortality risk may be partially explained by NAD^+^ levels. A variety of pathological conditions, such as CVD, obesity, and neurodegenerative diseases, are associated with dysregulation of cellular NAD^+^ levels.^34,35,36^ The basic requirement for NAD^+^ synthesis can be fulfilled by consuming less than 20 mg of daily niacin.^37^ Although the exact molecular mechanism of the association between dietary niacin intake and the risk of mortality among individuals with NAFLD requires further investigation, our findings align with existing evidence and are biologically plausible.

### Limitations

This study has limitations. First, the missing physical activity data were too many to perform multiple imputation and include the results in the adjustment model. We believe that the physical activity data are missing completely at random and that the resulting bias is minimal. Moreover, the E-value of 2.16 suggests that an unmeasured confounder must be associated with both the exposure (dietary niacin intake) and the outcome (mortality) with a relative risk greater than 2.16 to fully explain the current association. Thus, we believe that physical activity does not mask the existing association between niacin and risk of mortality. Second, as our study design is observational, we cannot establish causal relationships. Third, while the National Death Index is reliable in determining vital status, its ability to accurately classify CVD mortality is only modest.^38^ Fourth, although dietary data were obtained via two 24-hour dietary recalls, the existence of recall bias could not be completely ruled out. Additionally, it is possible that some potential confounders were not adequately accounted for, thereby allowing for the presence of residuals and unidentified confounders that cannot be entirely excluded.

## Conclusions

Findings from this cohort study of US adults with NAFLD suggest that higher dietary niacin intake may be associated with a lower risk of all-cause mortality among individuals with NAFLD. Higher dietary niacin intake was not found to be associated with a lower risk of CVD mortality in this study. The dose-response association of dietary niacin intake with reducing the risk of all-cause and CVD mortality among patients with NAFLD needs to be further investigated to determine optimal intake levels.

## Notes

Supplement 1. **eTable 1.** Healthy Eating Index-2015 components and scoring standards **eTable 2.** Associations of dietary niacin intake with CVD mortality in various subgroups among participants with NAFLD in NHANES 2003-2018 **eTable 3.** Associations of dietary niacin intake with all-cause mortality in subgroups of other B vitamins **eTable 4.** Associations of dietary niacin intake with all-cause mortality in subgroups of FIB-4 levels **eTable 5.** Association of dietary niacin intake with mortality after excluding NAFLD participants who died within 2 years of follow-up **eTable 6.** Association of dietary niacin intake with mortality after excluding NAFLD participants with only one dietary recall **eTable 7.** Association of dietary niacin intake with mortality after excluding NAFLD participants who died within 2 years of follow-up and had only one dietary recall **eFigure 1.** Association of dietary niacin intake with all-cause and CVD mortality among individuals with NAFLD as assessed using restricted cubic spline models with 3 knots **eFigure 2.** Association of dietary niacin intake with all-cause and CVD mortality among individuals with NAFLD as assessed using restricted cubic spline models with 5 knots

Supplement 2.
Data Sharing Statement
