# Supplementary material for: Dietary Niacin Intake and Mortality Among Individuals With Nonalcoholic Fatty Liver Disease
Source: JAMA Netw Open. 2024 Feb 1;7(2):e2354277. doi: 10.1001/jamanetworkopen.2023.54277 (PMC10835509; doi:10.1001/jamanetworkopen.2023.54277)
Supplement: Supplement 1. — eTable 1. Healthy Eating Index-2015 components and scoring standards eTable 2. Associations of dietary niacin intake with CVD mortality in various subgroups among participants with NAFLD in NHANES 2003-2018 eTable 3. Associations of dietary niacin intake with all-cause mortality in subgroups of other B vitamins eTable 4. Associations of dietary niacin intake with all-cause mortality in subgroups of FIB-4 levels eTable 5. Association of dietary niacin intake with mortality after excluding NAFLD participants who died within 2 years of follow-up eTable 6. Association of dietary niacin intake with mortality after excluding NAFLD participants with only one dietary recall eTable 7. Association of dietary niacin intake with mortality after excluding NAFLD participants who died within 2 years of follow-up and had only one dietary recall eFigure 1. Association of dietary niacin intake with all-cause and CVD mortality among individuals with NAFLD as assessed using restricted cubic spline models with 3 knots eFigure 2. Association of dietary niacin intake with all-cause and CVD mortality among individuals with NAFLD as assessed using restricted cubic spline models with 5 knots [file jamanetwopen-e2354277-s001.pdf]

Supplementary Online Content

Pan J, Zhou Y, Pang N, Yang L. Dietary niacin intake and mortality among individuals with nonalcoholic fatty liver disease. *JAMA Netw Open*. 2024;7(2):e2354277. doi:10.1001/jamanetworkopen.2023.54277

eTable 1. Healthy Eating Index-2015 components and scoring standards
eTable 2. Associations of dietary niacin intake with CVD mortality in various subgroups among participants with NAFLD in NHANES 2003-2018
eTable 3. Associations of dietary niacin intake with all-cause mortality in subgroups of other B vitamins
eTable 4. Associations of dietary niacin intake with all-cause mortality in subgroups of FIB-4 levels
eTable 5. Association of dietary niacin intake with mortality after excluding NAFLD participants who died within 2 years of follow-up
eTable 6. Association of dietary niacin intake with mortality after excluding NAFLD participants with only one dietary recall
eTable 7. Association of dietary niacin intake with mortality after excluding NAFLD participants who died within 2 years of follow-up and had only one dietary recall
eFigure 1. Association of dietary niacin intake with all-cause and CVD mortality among individuals with NAFLD as assessed using restricted cubic spline models with 3 knots
eFigure 2. Association of dietary niacin intake with all-cause and CVD mortality among individuals with NAFLD as assessed using restricted cubic spline models with 5 knots

This supplementary material has been provided by the authors to give readers additional information about their work.

eTable 1. Healthy Eating Index-2015 components and scoring standards[a]

Component	Maximum points	Standard for maximum score	Standard for minimum score of zero
**Adequacy**			
Total Fruits[b]	5	≥0.8 cup equiv. per 1,000 kcal	No Fruits
Whole Fruits[c]	5	≥0.4 cup equiv. per 1,000 kcal	No Whole Fruits
Total Vegetables[d]	5	≥1.1 cup equiv. per 1,000 kcal	No Vegetables
Greens and Beans[d]	5	≥0.2 cup equiv. per 1,000 kcal	No Dark Green Vegetables or Legumes
Whole Grains	10	≥1.5 oz equiv. per 1,000 kcal	No Whole Grains
Dairy[e]	10	≥1.3 cup equiv. per 1,000 kcal	No Dairy
Total Protein Foods[f]	5	≥2.5 oz equiv. per 1,000 kcal	No Protein Foods
Seafood and Plant Proteins[f, g]	5	≥0.8 oz equiv. per 1,000 kcal	No Seafood or Plant Proteins
Fatty Acids[g]	10	(PUFAs + MUFAs)/SFAs ≥2.5	(PUFAs + MUFAs)/SFAs ≤1.2
**Moderation**			
Refined Grains	10	≤1.8 oz equiv. per 1,000 kcal	≥4.3 oz equiv. per 1,000 kcal
Sodium	10	≤1.1 gram per 1,000 kcal	≥2.0 grams per 1,000 kcal
Added Sugars	10	≤6.5% of energy	≥26% of energy
Saturated Fats	10	≤8% of energy	≥16% of energy

[a] Intakes between the minimum and maximum standards are scored proportionately.

[b] Includes 100% fruit juice.

[c] Includes all forms except juice.

[d] Includes legumes (beans and peas).

[e] Includes all milk products, such as fluid milk, yogurt, and cheese, and fortified soy beverages.

[f] Includes seafood, nuts, seeds, soy products (other than beverages), and beans, peas, and lentils.

[g] Ratio of poly- and monounsaturated fatty acids (PUFAs and MUFAs) to saturated fatty acids (SFAs).

© 2024 Pan J et al. JAMA Network Open.

eTable 2. Associations of dietary niacin intake with CVD mortality in various subgroups among participants with NAFLD in NHANES 2003-2018

	Hazard ratio (95% CI) by tertile[a]			
	Dietary niacin intake, mg			
**Characteristic**	**T1 ≤18.4**	**T2 18.5-26.6**	**T3 ≥26.7**	***P* interaction**
Age,				
≤39	1.00 (Ref)	0.07 (0.00, 1.29)	0.37 (0.04, 3.92)	.16
40-59	1.00 (Ref)	1.19 (0.37, 3.82)	1.17 (0.32, 4.27)	
≥60	1.00 (Ref)	0.91 (0.61, 1.34)	0.53 (0.27, 1.04)	
Sex				
Male	1.00 (Ref)	1.03 (0.61, 1.71)	0.58 (0.26, 1.29)	.42
Female	1.00 (Ref)	0.75 (0.36, 1.58)	1.35 (0.61, 2.98)	
Race				
Mexican American	1.00 (Ref)	1.12 (0.35, 3.64)	0.31 (0.04, 2.34)	.66
Non-Hispanic Black	1.00 (Ref)	0.51 (0.16, 1.67)	0.44 (0.13, 1.49)	
Non-Hispanic White	1.00 (Ref)	0.77 (0.53, 1.13)	0.61 (0.29, 1.26)	
Other[b]	1.00 (Ref)	3.43 (1.14, 10.4)	2.99 (0.71, 12.5)	
Education levels				
<High school	1.00 (Ref)	1.21 (0.61, 2.39)	0.97 (0.33, 2.82)	.12
High school	1.00 (Ref)	1.11 (0.47, 2.62)	1.66 (0.74, 3.75)	
Some college or above	1.00 (Ref)	0.47 (0.24, 0.92)	0.19 (0.08, 0.44)	
Family income–poverty ratio				
<1.0	1.00 (Ref)	0.73 (0.29, 1.88)	1.59 (0.50, 5.05)	.18
1.0-3.0	1.00 (Ref)	0.64 (0.38, 1.07)	0.55 (0.29, 1.05)	
>3.0	1.00 (Ref)	1.85 (0.68, 5.02)	0.89 (0.18, 4.27)	
Smoking status				
Never smoker	1.00 (Ref)	1.16 (0.57, 2.37)	1.37 (0.64, 2.95)	.12

© 2024 Pan J et al. JAMA Network Open.

Former smoker	1.00 (Ref)	0.63 (0.35, 1.12)	0.56 (0.24, 1.32)	
Current smoker	1.00 (Ref)	1.50 (0.38, 5.89)	0.20 (0.03, 1.43)	
BMI				
<25.0	1.00 (Ref)	1.33 (0.23, 7.68)	0.00 (0.00, 0.00)	.09
25.0-29.9	1.00 (Ref)	2.05 (0.73, 5.72)	0.58 (0.13, 2.68)	
≥30.0	1.00 (Ref)	0.74 (0.50, 1.11)	0.75 (0.40, 1.42)	
Diabetes				
Yes	1.00 (Ref)	0.97 (0.49, 1.92)	0.68 (0.24, 1.92)	.95
No	1.00 (Ref)	0.84 (0.56, 1.27)	0.63 (0.24, 1.65)	

Abbreviations: BMI, body mass index; NAFLD, Nonalcoholic fatty liver disease; NHANES, National Health and Nutrition Examination Survey.

[a] Adjusted for age, sex, race and ethnicity, education levels, family income–poverty ratio, smoking status, BMI, total energy intake, diabetes, hypertension, dyslipidemia, use of dietary supplements, HDL/TC, and HEI-2015. The strata variable was not included when stratifying by itself.

© 2024 Pan J et al. *JAMA Network Open*.

eTable 3. Associations of dietary niacin intake with all-cause mortality in subgroups of other B vitamins

Characteristic	Hazard ratio (95% CI) by tertile[a] Dietary niacin intake, mg			*P* interaction
	**T1 ≤18.4**	**T2 18.5-26.6**	**T3 ≥26.7**	
Vitamin B1, mg				
<1.4	1.00 (Ref)	0.80 (0.55, 1.17)	0.56 (0.31, 1.02)	.33
≥1.4	1.00 (Ref)	0.84 (0.51, 1.40)	0.77 (0.45, 1.32)	
Vitamin B2, mg				
<1.8	1.00 (Ref)	0.86 (0.57, 1.29)	0.57 (0.31, 1.04)	.25
≥1.8	1.00 (Ref)	0.84 (0.51, 1.40)	0.77 (0.45, 1.32)	
Vitamin B6, mg				
<1.7	1.00 (Ref)	0.81 (0.59, 1.12)	0.26 (0.10, 0.71)	.02
≥1.7	1.00 (Ref)	0.95 (0.55, 1.62)	0.95 (0.59, 1.55)	
Vitamin B9, mcg				
<186.0	1.00 (Ref)	0.80 (0.57, 1.14)	0.56 (0.35, 0.89)	.26
≥186.0	1.00 (Ref)	0.89 (0.56, 1.42)	0.85 (0.52, 1.39)	
Vitamin B12, mcg				
<4.0	1.00 (Ref)	0.68 (0.47, 0.99)	0.53 (0.30, 0.93)	.52
≥4.0	1.00 (Ref)	0.85 (0.57, 1.27)	0.80 (0.55, 1.18)	

All other B vitamins were dichotomized according to their median levels in the study population.

a Adjusted for age, sex, race and ethnicity, education levels, family income–poverty ratio, smoking status, BMI, total energy intake, diabetes, hypertension, dyslipidemia, use of dietary supplements, HDL/TC, and HEI-2015.

© 2024 Pan J et al. JAMA Network Open.

eTable 4. Associations of dietary niacin intake with all-cause mortality in subgroups of FIB-4 levels

Characteristic	Hazard ratio (95% CI) by tertile[a] Dietary niacin intake, mg T1 ≤18.4	T2 18.5-26.6	T3 ≥26.7	*P* interaction
FIB-4				
<1.30	1.00 (Ref)	0.84 (0.52, 1.36)	0.64 (0.39, 1.07)	.25
≥1.30	1.00 (Ref)	0.73 (0.52, 1.04)	0.79 (0.50, 1.23)	
FIB-4				
<2.67	1.00 (Ref)	0.80 (0.60, 1.07)	0.70 (0.50, 0.99)	.13
≥2.67	1.00 (Ref)	0.47 (0.18, 1.20)	0.77 (0.23, 2.54)	

a Adjusted for age, sex, race and ethnicity, education levels, family income–poverty ratio, smoking status, BMI, total energy intake, diabetes, hypertension, dyslipidemia, use of dietary supplements, HDL/TC, and HEI-2015.

© 2024 Pan J et al. JAMA Network Open.

eTable 5. Association of dietary niacin intake with mortality after excluding NAFLD participants who died within 2 years of follow-up

	Hazard ratio (95% CI)			
	Dietary niacin intake, mg			
Model	T1 ≤18.5	T2 18.6-26.6	T3 ≥26.7	*P* trend
**All-cause mortality**				
No. deaths/total	204/1418	151/1400	112/1418	
Model 1[a]	1.00 (Ref)	0.77 (0.59, 1.01)	0.48 (0.36, 0.63)	<.001
Model 2[b]	1.00 (Ref)	0.77 (0.60, 1.00)	0.55 (0.39, 0.79)	.001
Model 3[c]	1.00 (Ref)	0.85 (0.64, 1.12)	0.73 (0.50, 1.07)	.11
**CVD mortality**				
No. deaths/total	79/1418	54/1400	33/1418	
Model 1[a]	1.00 (Ref)	0.86 (0.63, 1.19)	0.34 (0.20, 0.58)	<.001
Model 2[b]	1.00 (Ref)	0.93 (0.67, 1.28)	0.46 (0.26, 0.83)	.007
Model 3[c]	1.00 (Ref)	1.02 (0.72, 1.45)	0.62 (0.31, 1.22)	.16

[a] Crude model.

[b] Adjusted for age and sex.

[c] Further adjusted for race and ethnicity, education levels, family income–poverty ratio, smoking status, BMI, total energy intake, diabetes, hypertension, dyslipidemia, use of dietary supplements, HDL/TC, and HEI-2015.

© 2024 Pan J et al. JAMA Network Open.

eTable 6. Association of dietary niacin intake with mortality after excluding NAFLD participants with only one dietary recall

Model	Hazard ratio (95% CI) Dietary niacin intake, mg T1 ≤18.4	T2 18.5-26.5	T3 ≥26.6	*P* trend
**All-cause mortality**				
No. deaths/total	221/1274	156/1286	119/1284	
Model 1[a]	1.00 (Ref)	0.67 (0.50, 0.90)	0.45 (0.33, 0.61)	<.001
Model 2[b]	1.00 (Ref)	0.63 (0.47, 0.84)	0.46 (0.32, 0.65)	<.001
Model 3[c]	1.00 (Ref)	0.74 (0.54, 1.01)	0.65 (0.45, 0.92)	.02
**CVD mortality**				
No. deaths/total	85/1274	59/1286	31/1284	
Model 1[a]	1.00 (Ref)	0.72 (0.51, 1.01)	0.42 (0.21, 0.83)	.01
Model 2[b]	1.00 (Ref)	0.69 (0.49, 0.98)	0.45 (0.24, 0.84)	.01
Model 3[c]	1.00 (Ref)	0.81 (0.57, 1.15)	0.62 (0.33, 1.14)	.13

[a] Crude model.

[b] Adjusted for age and sex.

[c] Further adjusted for race and ethnicity, education levels, family income–poverty ratio, smoking status, BMI, total energy intake, diabetes, hypertension, dyslipidemia, use of dietary supplements, HDL/TC, and HEI-2015.

© 2024 Pan J et al. JAMA Network Open.

eTable 7. Association of dietary niacin intake with mortality after excluding NAFLD participants who died within 2 years of follow-up and had only one dietary recall

	**Hazard ratio (95% CI)**			
	**Dietary niacin intake, mg**			
**Model**	**T1 ≤18.5**	**T2 18.6-26.6**	**T3 ≥26.7**	***P* trend**
**All-cause mortality**				
No. deaths/total	189/1258	136/1260	103/1258	
Model 1[a]	1.00 (Ref)	0.69 (0.52, 0.93)	0.45 (0.33, 0.61)	<.001
Model 2[b]	1.00 (Ref)	0.64 (0.48, 0.85)	0.46 (0.33, 0.66)	<.001
Model 3[c]	1.00 (Ref)	0.76 (0.55, 1.05)	0.67 (0.44, 1.00)	0.06
**CVD mortality**				
No. deaths/total	71/1258	50/1260	28/1258	
Model 1[a]	1.00 (Ref)	0.73 (0.51, 1.03)	0.33 (0.18, 0.61)	<.001
Model 2[b]	1.00 (Ref)	0.71 (0.50, 1.02)	0.38 (0.21, 0.72)	0.002
Model 3[c]	1.00 (Ref)	0.85 (0.56, 1.30)	0.56 (0.28, 1.15)	0.11

[a] Crude model.

[b] Adjusted for age and sex.

[c] Further adjusted for race and ethnicity, education levels, family income–poverty ratio, smoking status, BMI, total energy intake, diabetes, hypertension, dyslipidemia, use of dietary supplements, HDL/TC, and HEI-2015.

© 2024 Pan J et al. JAMA Network Open.

eFigure 1. Association of dietary niacin intake with all-cause and CVD mortality among individuals with NAFLD as assessed using restricted cubic spline models with 3 knots

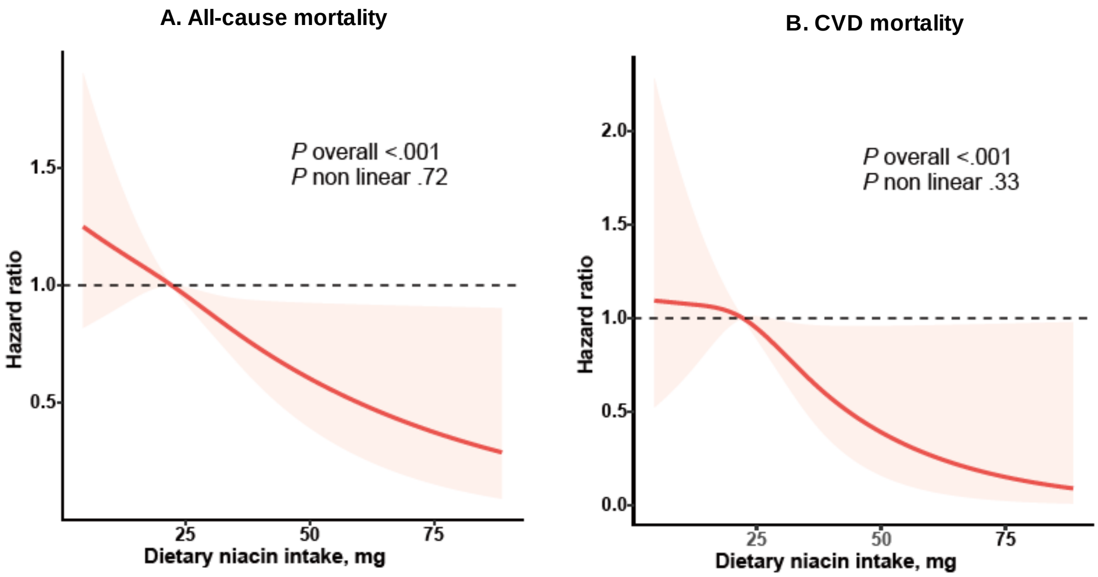

Abbreviations: CVD, cardiovascular disease; NAFLD, nonalcoholic fatty liver disease.
Hazard ratios were adjusted for age, sex, race and ethnicity, education levels, family income–poverty ratio, smoking status, BMI, total energy intake, diabetes, hypertension, dyslipidemia, dietary supplement, HDL/TC, and HEI-2015.
The shaded areas represent 95% confidence intervals of the hazard ratio.
Restricted cubic spline models with 3 knots (at the 10th, 50th, and 90th percentiles).

© 2024 Pan J et al. JAMA Network Open.

eFigure 2. Association of dietary niacin intake with all-cause and CVD mortality among individuals with NAFLD as assessed using restricted cubic spline models with 5 knots

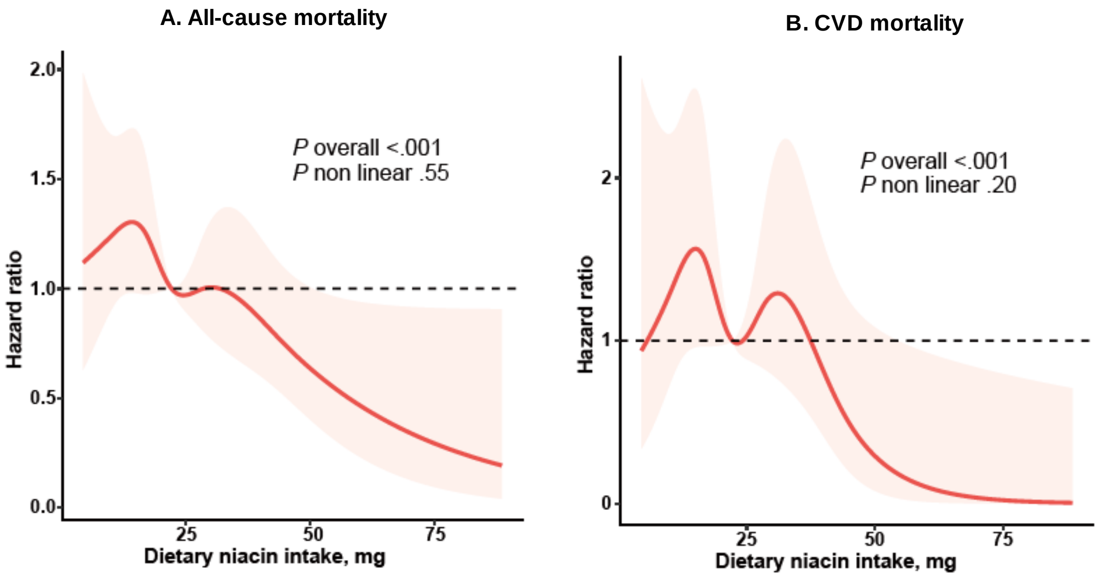

Abbreviations: CVD, cardiovascular disease; NAFLD, nonalcoholic fatty liver disease.

Hazard ratios were adjusted for age, sex, race and ethnicity, education levels, family income–poverty ratio, smoking status, BMI, total energy intake, diabetes, hypertension, dyslipidemia, dietary supplement, HDL/TC, and HEI-2015.

The shaded areas represent 95% confidence intervals of the hazard ratio.

Restricted cubic spline models with 5 knots (at the 5th, 27.5th, 50th, 72.5th, and 95th percentiles).

© 2024 Pan J et al. JAMA Network Open.
