# Supplementary material for: Dietary Niacin Intake and Mortality Among Individuals With Nonalcoholic Fatty Liver Disease
Source: JAMA Netw Open. 2024 Feb 1;7(2):e2354277. doi: 10.1001/jamanetworkopen.2023.54277 (PMC10835509; doi:10.1001/jamanetworkopen.2023.54277)
Supplement: Supplement 2. — Data Sharing Statement [file jamanetwopen-e2354277-s002.pdf]

Data Sharing Statement

Pan. Dietary Niacin Intake and Mortality Among Individuals With Nonalcoholic Fatty Liver Disease. *JAMA Netw Open*. Published February 01, 2024.
doi:10.1001/jamanetworkopen.2023.54277

Data
Data available: No

Additional Information
Explanation for why data not available: The data described in the manuscript, codebook, and analytic code will not be made available as it was obtained from the NHANES database, which is freely accessible to all researchers worldwide.
